# Codesign of Food System and Circular Economy Approaches for the Development of Livestock Feeds from Insect Larvae

**DOI:** 10.3390/foods10081701

**Published:** 2021-07-22

**Authors:** Sandeep Jagtap, Guillermo Garcia-Garcia, Linh Duong, Mark Swainson, Wayne Martindale

**Affiliations:** 1 Sustainable Manufacturing Systems Centre, Cranfield University, Cranfield MK43 0AL, UK; S.Z.Jagtap@cranfield.ac.uk; 2 Department of Chemical and Biological Engineering, The University of Sheffield, Sheffield S1 3JD, UK; g.garcia-garcia@sheffield.ac.uk; 3 Faculty of Business and Law, The University of the West of England, Bristol BS16 1QY, UK; linh.duong@uwe.ac.uk; 4 National Centre for Food Manufacturing, University of Lincoln, Holbeach PE12 7PT, UK; mswainson@lincoln.ac.uk

**Keywords:** black soldier fly larvae, circular economy, food waste, poultry, aquaculture, waste valorisation

## Abstract

Processes that utilise low-value wastes and convert them to high-value food ingredients systemically add value across commercial operations. Current common disposal options include use as animal feed, anaerobic digestion, composting, incineration, and the worst-case options of landfill and wastewater disposal. The pressure is acute with food manufacturers needing to align with the UN Sustainable Development Goals and reach targets of zero waste to landfill. This research identifies black soldier fly larvae as a bioreactor that converts most food waste into high-value feed materials. Production of larvae and the regulatory framework for their use as animal feed is being assessed in several nations. The requirement to understand the availability of feedstocks for larvae production and the capability to establish feedstock supply chains was tested in this study using geographical information system and life cycle assessment methodologies, providing new research insights for resource utilisation in a circular economy.

## 1. Introduction

The agri-food sector is facing significant challenges as the world population is expected to grow by 2.3 billion (i.e., one-third of the current population (7.8 billion) by 2050), which means that agricultural and food production will have to increase to meet demand [1]. This challenge will be compounded by the impacts of climate change [2]. While agricultural and food processing is attuned to increasing production efficiency through sustainable intensification, it is also responsible for the global impact on ecosystem services including loss of soils, increased greenhouse gas (GHG) emissions, and loss of biodiversity [3]. The global demand for livestock feeds as well as their prices has increased over the period of the COVID-19 pandemic [4], particularly for limited options of products derived from maize, wheat, soy, and dairy supply [5]. The assurance associated with feed materials internationally is also under increased scrutiny concerning safety, and contamination with mycotoxins in crops recalled for human consumption are diverted into feed supply chains. Toxins such as aflatoxins and mycotoxins are not the only assurance issue that is spotlighted with feed because the use of genetically modified (GM) feeds needing to be removed from GM-free supply chains by regulation in specific regions [6]. The requirement for feed materials to meet assurance requirements is of acute concern for the global food system. The localised production of black soldier fly larvae (BSFL) in facilities within line of sight of feedstock production offers enhanced assurance opportunities and the capability to do this is tested in the reported research. 

**Figure 1:**
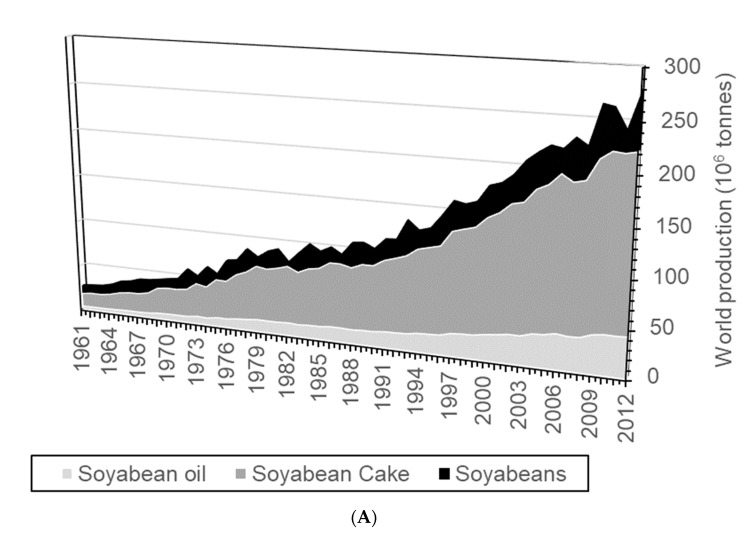

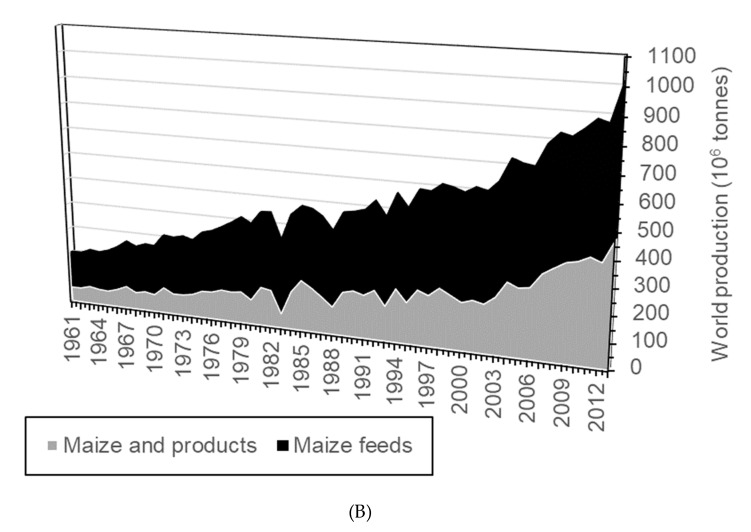
(**A**) The global production of soybean products and their use as feed 1961–2012 [7]. (**B**) The global production of maize products and their use as feed 1961–2012 [7].

Global feed demand is increasing, and this is indicated by the global production of maize and soybeans, whose main products are crucial feeds (Figure 1). Even within these large-scale feed and food crop supply chains, there is much potential to recycle wastes by conversion to insect protein produced locally at points of food waste generation. The global level of waste in these supply chains are low in terms of total production, but significant in terms of volume with global soybean losses at 2% of 278 × 10^6^ tonnes produced in 2013 and maize product global losses at 4% of 1.02 × 10^9^ tonnes produced. The development of localised conversion of in-field crop losses such as these to high-value animal feed as BSFL offers the potential to develop a circular economy that approaches zero waste.

Figure 1A,B shows the global demand for the top global plant protein feeds are increasing, even though the segmentation of products from them is innovative in that they are highly diversified, providing ingredients and fine chemicals such as oils, starches, and nutraceuticals. The extension of these products into different markets means that they require greater resilience in a global market where their demand is increasing. An important option to build resilience to the growing feed markets is to define alternative feed sources, and this has been in part achieved using whey products and fish meals. Still, even these markets are experiencing restrictions for feeds. The fishmeal feed supply has always been limited to 1.5–2.0 × 10^6^ tonnes yr^−1^ because of the requirements of sustainable fisheries management. Indeed, the issue of sustainable harvesting of natural resources was perhaps first highlighted by the exploitation of anchoveta fisheries for fishmeal production. The fisheries controversy is embedded in the development of natural resource management practices and the development of food certifications. The use of whey as feed has an established history, but there are different pressures in that whey production is greater at 113 × 10^6^ tonnes yr^−1^ and the development and valorisation of whey as a food ingredient has diverted volume from feed to human consumption as a supplement and food [8].

**Figure 2:**
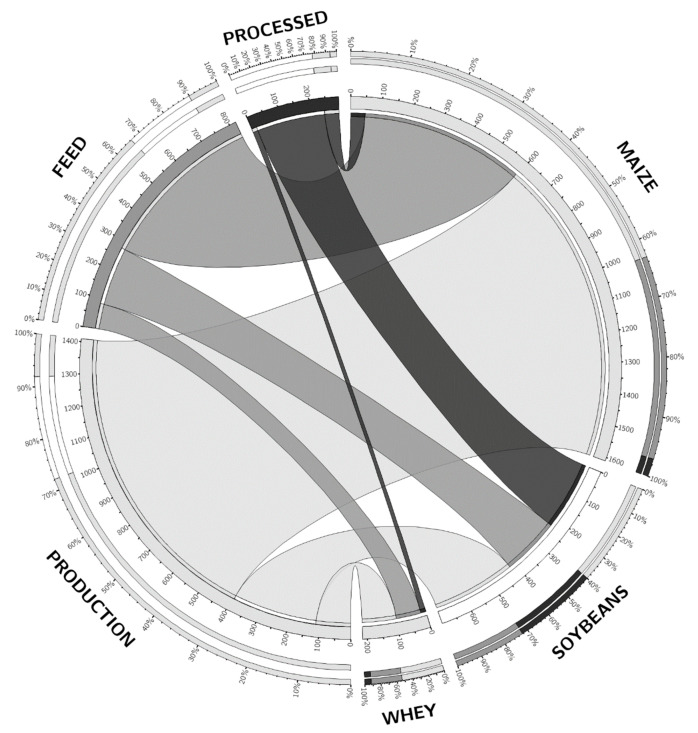
The segmentation of maize, soybean, and whey volume into feed and processing supply chains. The inner scale of the synteny diagram show the accumulated mass of product in million tonnes produced each year, the outer scale shows relative percentage of production, processing, and feed [7,10]. The synteny diagram has been developed using the open source Circos on-line programme [10].

The processing of crop biomass in higher value ingredients is important because supply chains will grow and operate commercially when value can be increased. Where waste has low value, there tends to be a race to the bottom of the value chain unless convenient conversion processes are available at the point of production of wastes. This principle is demonstrated in Figure 2, in that the amount of feed available globally is dominated by maize where 60% is not processed into higher-value ingredients to the scale of soybeans milled for oil extraction before being used in feeds as soy meal. In whey processing, water removal provides high value whey powders, and it is these conversion processes that are crucial so that utilising a low-cost bioreactor model such as BSFL is opportune if the supply of wastes and convertors can be connected efficiently with relevant separation technologies for biomolecules [9]. The differences between the soybean, maize, and whey supply and processing systems also demonstrate how waste reduction and valorisation processes need to be generically applied across different feedstock supply chains, resulting in the value-added production of ingredients to be used as food or feed.

The production through to feed and processing operations for the commodities shown in Figure 2 demonstrate the value of providing a farm-to-fork analysis of the value chain because of the volumes of feedstock utilised. There is demand for feed and the production of varied waste quantities such as in Europe, where 700 million tonnes of agricultural waste are annually available, can provide feedstock [11]. Implementing a circular economy becomes essential to address the wastes generated, and this will require a system-based valorisation process for biomass-to-feed supply chains. The feeds produced must obtain maximum value from feedstocks, and BSFL production has been identified as a method for doing this. This is because the production of BSFL can be mobilised so that production units can be placed at the site of either feedstock production for feeding BSFL or sites of feed use by livestock. Hence, the BSFL production strategy is of dual purpose to conserve material resources by reducing waste and enhancing the resource efficiency path to provide a localised and circular economy [12]. Researchers have defined waste valorisation and often considered the preferred approach to treat food waste even though the means to conversion for commercial operators are rarely reported [13,14,15]. Food waste valorisation can be defined as any activity that includes converting food waste into useful products or extracting useful compounds. These options have been demonstrated to show potential environmental, social, and economic advantages.

Food wastes from processing and manufacturing have been used as feedstocks for the production of BSFL and their conversion of low-value biomass into a high-quality protein has been tested. BSFL is an important candidate species because their demonstrators have shown 3.7–11.8% bioconversion of feedstock wet weight during 15–52-day BSFL developmental periods [16]. The bioconversion of feedstocks results in 32–58% protein and 18–39% lipid in the dry weight of larvae where waste reduction of 38–55% wet weight and 26–72% dry weight is achievable. The value of nutrients to nutrition is critical here, where the bioavailability of nutrients from non-plant sources are favourable and insect proteins may offer solutions to poor bioavailability [17]. BSFL will feed and convert 3.7–11.8% of feedstock to larval biomass with a significant reduction in biowaste due to metabolic activity during bioconversion to protein, carbohydrate, and fats, where BSFL provide efficient bioreactor units [18]. The bioconversion activity demonstrated for manures and food industry biowastes demonstrates that high-quality protein recovery is possible [19]. The use of BSFL in aquaculture has increased the demand for these feeds as they replace existing fish-meals obtained from pelagic species where supply is both environmentally sensitive and volatile. The national post-farm gate food waste inventory for the UK is 9.5 million tonnes generated in 2018, which includes waste arising from domestic (6.6 × 10^6^ tonnes, 69%), manufacturing (1.5 × 10^6^ tonnes, 16%), foodservice (1.1 × 10^6^ tonnes, 12%); and retail (0.3 × 10^6^ tonnes, 3%) [20]. The direct economic value of this is placed over £GBP 19 billion and produces up to 25 million tonnes of GHG emissions. This type of national food waste inventory approach has meant that the low-value status of food waste must change. Providing innovations such as BSFL production that can convert these materials into high value feeds offers a route to increasing productivity, and economically ameliorate GHG emissions. The UK Government has already charted a route to carbon zero targets, and in the case of agriculture and food, waste reduction is a high-profile route to decreasing GHG emissions to a residual emission baseline or carbon zero outcome. The approach also reduces the so-called Scope 3 GHG emissions associated with supply chain activities such as food losses and wastes. Scope 3 GHG emissions reduction is difficult to quantify because of their variability in supply chain operations. The production of BSFL in a circular economy provides a specific means of measuring and quantifying Scope 3 emission reduction by converting what would be wasted into high value feeds. 

The research reported here demonstrates how utilisation of food wastes as feedstock for BSFL production reduces GHG emissions and provides the potential to localise feed production in a circular economy. The production of feed is located and co-designed for livestock production activities so that the outcomes have important environmental and assurance benefits. The assessment reported here is for the quantification of feedstocks from agri-production and manufacturing operations in food supply chains. The mass of feedstocks required for BSFL production needs to be sufficient for the existing circular economy systems that include waste-to-energy schemes (e.g., anaerobic digestion, incineration) and composting. Any future co-design of BSFL production into these circular economies will embed a diversion of feedstocks from landfill options. These actions will be quantifiable because they remove the system’s waste mass and transform it into a high-value feed. Therefore, it will align to targets guided by the UN Sustainable Development Goals (SDGs) [21]. This research presents four key drivers for these changes: (a) demand and economic development; (b) social and ethical; (c) innovation and Industry 4.0; and (d) alignment to the UN SDGs.

### 1.1. Demand and Economic Development Drivers for BSFL Production 

The global animal feed market is projected by business services to generate revenues of £483 × 10^9^ by 2023 [22]. It is 7% of the total global food system revenue that currently approaches £7 × 10^12^, so feed, and the resources to produce it are of significant economic value. The increase in demand is demonstrated in Figure 1, where highly developed segmented maize and soybean ingredient markets support efficient, globally distributed feed supply. The global increase in meat and dairy consumption is the source of the demand for quality feeds, with dairy and poultry protein typically being in the top-three ranked protein sources for many nations [23]. The increase in feed demand is associated with livestock product demand, even though there is evidence of meat consumption stabilising or even decreasing in specific global regions such as Europe [24]. The supply of stable and resilient feed protein supplies that either replace or supplement the current food supply is important for developing a robust global food system, even if livestock product consumption stabilises or decreases. The supply of the main feed materials globally shows a year-on-year increase in supply. There is an indication that the volatility of maintaining these fails to meet demand (Figure 1A,B). The relationship is not just based on quantity, because the feeds’ protein and oil contents are critical in feeds supplying poly-unsaturated fatty acids. BSFL notably contains favourable protein and oil profiles proven for the production of poultry and eggs [25]. The favourable economic outcomes of BSFL production are notable with regard to conservation of land use, where livestock production is projected to utilise 20% more arable land of current use by 2050 [26]. The life cycle assessment (LCA) of BSFL production demonstrates land-use requirements are dependent on the feedstock used to produce the BSFL, even though the BSFL production units require minimal land resources [27]. The dependence of BSFL production on feedstocks that are variable means it is essential to utilise BSFL as an alternative source of animal feed that supplements existing feed supplies and provides an impetus to developing a circular economy where variable feedstocks are used for protein and oil bioconversion. The resource use balance of BSFL production is apparent, but dependent on feedstock; the economic challenge is to understand where feedstocks are produced so that BSFL production can be developed in optimal locations to promote a sustainable circular economy.

### 1.2. Social and Ethical Drivers for BSFL Production 

Consumers’ social and ethical expectations of food products are components of the quantification of sustainable lifestyles, with media spotlighting specific food systems such as those from soybean being associated with land-use change and deforestation. A call for a social change agenda embedded in the food system for sustainable outcomes is evident in the alignment of practices to the SDGs in food and beverage companies [21]. Changes in consumer preferences for sustainably farmed meat and seafood products have seen alignment to stringent animal welfare standards where feeding systems that are stable, efficient, and associated with the circular economy will be favoured. These requirements go beyond those needed to qualify nutritional quality with the example of the emergent demand for non-genetically engineered (GE) soybean feeds, providing an impetus to qualify feed provenance. The requirements for non-GE feed move beyond those requirements for legal declarations of safety because at least half of the globally traded soybean crop and by extension soy meal, cake, and oil derivatives are from herbicide-resistant crop varieties that have been genetically engineered. Localising the supply of feed using BSFL will alleviate the pressures placed on those supply chains demanding feeds from non-GE sources with reduced land-use change impacts. The localisation of feed production will change the food systems’ impact because 26% of the world’s total greenhouse gas emissions are agricultural sources and 6% arise from growing the crops for animal feed [28]. Developing viable, clean, and green technological solutions for reducing the environmental impact associated with animal feed is of interest to livestock production sectors and future food security policy. 

### 1.3. Innovation and Industry 4.0 for BSFL Production 

Industry 4.0 includes every specialised means to deal with data, optimise productivity [29], and as such, it can increase the efficiency of the feed supply chain in order to support the future animal feed demand. Industry 4.0 applications reduce labour costs by stimulating the development of the BSFL production factory in large-scale insect-protein production. Scaling to industrial production has been tested, and these are shown in Table 1, which leads us to identify the more important challenges of localising processing options and enhancing connectivity across BSFL supply chains so that circular economies can develop [30,31]. The application of Industry 4.0 including the Internet of Things (IoT), robotics and automation, and big data analytics will enable data collection for assurance, analysis for quality, control for improved productivity, and better-informed decision-making with regard to the optimisation of the BSFL production [32,33]. Adopting an Industry 4.0 approach will deliver less resource wastage, and automated processes such as temperature control could increase the BSFL productivity in processing the food waste. Artificial intelligence and robotics will be used to identify optimal rations of feedstocks, harvesting periods, and automate the BSF breeding process, respectively [34]. 

### 1.4. Feed and Food Supply Chain Wastes 

The supply chain losses of feed and food materials are significant and are typically below 5–10% of production volumes so that the actual volumes of potential BSFL feedstock are significant. The outlook for BSFL protein providing a sustainable feed source is promising when compared to other livestock derived feeds [35]. The processes required for BSFL production include the supply of fresh larvae and processed meals that are dried and milled. These processes enable the concentration of nutrients by weight or the development of feedstocks that can be segregated by separation into protein and fat preparations for ingredients. Pilot-scale production of pupae and concentrated meals have been tested using LCA to show that insect meals are favourable when compared to whey, egg protein, and fishmeal feed products [35]. Smetana et al. [35] applied attributional LCA to demonstrate the benefits of using co-products as a feedstock for BSF production that would have otherwise remained unutilised and lost together with the value of co-products such as fertiliser from BSFL production. The tests also used a consequential LCA to assess the benefit of using feed replacement from BSFL production. The models developed identified how, over long-term periods of up to 10 years, the production of insect meals and their production impacts can be reduced to less than those of soy-based feeds, if recycled energy is used to dry, mill, and separate insect meals. In a previous study, Smetana et al. [27] concluded that the use of insect-based feed produced at an industrial level could reduce environmental impacts compared to conventional feeds. The transport of BSFL and insect-meal from the sites of production to their use in feed systems provides an opportunity in that if production is at the site of co-product or livestock production, it would reduce the environmental impacts.

### 1.5. Assessment of the UK Food System for BSFL Feed Supply 

The annual post-farm gate food waste arising in UK households, hospitality and foodservice, food manufacture, retail and wholesale sectors in 2018 was 9.5 million tonnes [20]. This study focused on the 1.5 million tonnes generated by food manufacturers. The potential to hyper-localise BSFL production to tackle domestic food waste exists. Such hyper-localised solutions have been tested with regard to composting programmes and the collection of green waste from residential areas. It should be recognised that such collection schemes are expensive, and if the food waste hierarchy of preferential solutions to reducing food waste is to be acted on, the localisation of bioconversion must be considered [36]. The UK imported £GBP 2.3 billion animal feed in 2018 [37], where feed cereals accounted for 3.9 million tonnes from non-EU nations and 2.2 million tonnes from EU nations. The 13.9 million tonnes of cereal supplied as feed for cattle, pigs, poultry, and sheep indicates a trading complexity that introduces low resilience and potential for volatility in supply [38]. BSFL production has been tested across several studies that have identified candidate feedstocks for BSFL production and products from BSFL preparations. A total replacement of fish meal with BSFL meal in the diets of sea-water Atlantic salmon has been demonstrated for growth performance, feed utilisation, nutrient digestibility, and the finished fillets sensory attributes [39]. Aquaculture is an important market for BSFL derived feeds, and the benefits of using BSFL feeds has been demonstrated across other popular fish species [40]. The use of BSFL products in the feeding of birds is of great interest because of the demand for poultry by consumers globally, and BSFL has been tested and demonstrated [41].

### 1.6. Proposed BSF Production Systems

**Figure 3:**
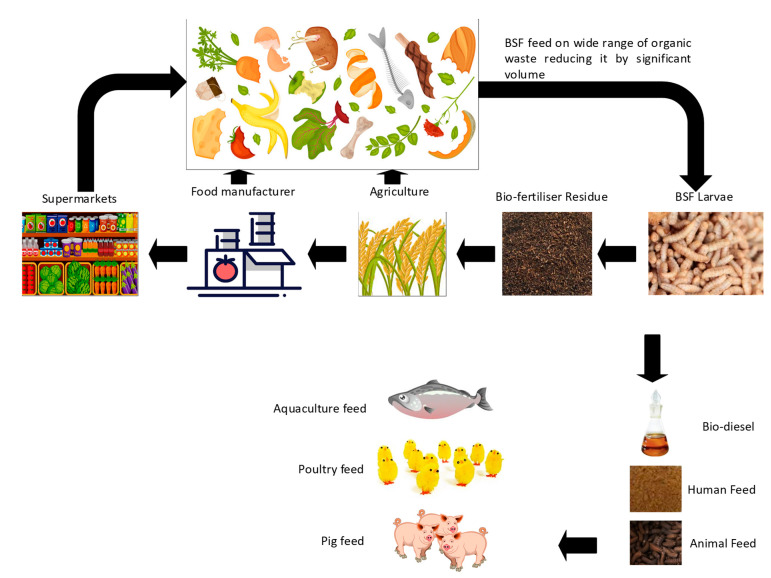
A schematic of BSFL circular economy utilising food manufacturing waste.

In this research, we proposed BSFL be used as an animal feed for poultry and aquaculture industries where food supply chain waste generated from the UK food manufacturers is easy to collect at low cost, and these systems were tested for poultry [42]. The collected food waste is transported to a bioconversion site that can be localised, where waste is converted to BSFL. The process would generate BSFL to be used as animal feed and the residue to be used as a soil conditioner and plant nutrient. Walter et al. [43] demonstrates that utilises food manufacturing waste as a feedstock for BSFL production, as shown in Figure 3. This approach is tested using food waste data collected from 38 UK-based food manufacturers, as shown in the Supplementary Materials. The data show the total amount of food produced, the amount of food waste generated by each company, the time period when the food waste was generated, and what kind of food waste management practice was adopted. The preferred and easier route to obtain value from food waste is anaerobic digestion, where the location of processing sites is nationwide in the UK [44]. The biogas production facilities are classified as (1) agricultural plants that use agricultural feedstocks such as manures, slurries, crops, and crop residues; and (2) waste plants that use feedstock obtained from municipal, commercial, and industrial waste streams. The capacity end-use of heat and/or power (CHP) or biomethane to grid (BtG) is nationwide in the UK, producing, 0.2–0.7% of the UK’s energy requirements, and part of the EU Renewable Energy Directive requires that 15% of the energy is delivered to UK consumers. Most important for anaerobic digestion is the circular economy value in meeting the EU Waste Framework Directive, which requires 50% of household waste to be recycled by 2020. 

## 2. Materials and Methods

The research presented here uses geographic information systems (GISs) to present supply chain models and it identifies where resource volume can enable innovations that deliver sustainable feeds. The research has been developed using data derived from open-access datasets described here that have been geocoded, so that location modelling is made possible for resource planning and respective sustainability and security assessments. MapInfo 17.02 software was used to plot geographic data using Edina Agcensus services for the Agricultural and Horticultural Survey (AHS) at a 2 km^2^ grid resolution [45] and the fame business databases [46]. The AHS is geocoded every ten years and the latest data for this research was 2010, which was used to plot cereal production intensity. The fame database provides business postcodes that were geocoded to six figure grid references for geographical plots. The fame databases only provide the registered office geo-locations and those companies who publicly report their business information. It is within these limitations that the models presented in this research are made. 

The methodology used for the environmental impact analysis is a streamlined life cycle assessment, commonly used to assess the products and processes’ environmental impact over their life cycle. The functional unit used for both scenarios was the treatment of 1525 t day^−1^ of food waste, as this is the current amount of food waste sent to anaerobic digestion by the 38 food companies analysed in the previous section. The life cycle inventory, with all materials and processes considered in both scenarios, and the data sources used, can be seen in Table 2. Consequential data were used to model anaerobic digestion in order to consider the avoided heat, electricity, and fertiliser obtained from the biogas and digestate of the process. We applied the ReCiPe 2016 Midpoint (H) V1.03/World (2010) H/A method in the life cycle impact assessment phase to obtain the characterised results. We then used the ReCiPe 2016 Endpoint (H) V1.03/World (2010) H/A method to identify the best scenario overall.

Similarly, a consequential approach was used to model the alternative scenario, considering the reduction of conventional animal feed produced to feed hens and fishmeal, and the reduction of conventional compost production, as these are the three outputs provided by the alternative scenario. It was assumed that half of the animal feed from the BSFL meal would be used to feed poultry and the other half to feed fish. It was assumed that 59% of the food waste would be evaporated as a water emission (food waste typically has a water content of 80%). To this emission, it should be added 55% of the water dried from the insect meal obtained and 94 t from the water added as input. The yield of the different products was assumed as 11% wet insect meal (5% dry insect meal), 30% compost, and the rest being the aforementioned evaporated water [47] and air emissions [48]. The amount of dry insect meal and compost obtained was proportionally adjusted to account for the carbon dioxide and methane emissions.

## 3. Results and Discussion

Anaerobic digestion is the preferred route for recycling food waste, and the 38 UK-based food and drink manufacturers assessed showed that 62% of food waste is providing feedstocks for anaerobic digestion. Controlled combustion for energy generation and composting accounts for 13% and 8%, respectively, with the option of effluent release and land application accounting for 7% and 6%, respectively. Extremely low quantities are used for animal feed (1%) due to hygiene regulations and risk assessments associated with human health and zoonoses. The importance of diverting waste from landfill has been embedded in the food manufacturing sector, with 1% of waste going to landfill and 2% of food waste is associated with biomass that is not harvested. 

The total amount of food waste generated from the sample of 38 companies tested in this study was 560,187 tonnes, 37% of the total 1.5 × 10^6^ tonnes of food waste generated by UK food manufacturers each year. This sample of 38 companies can provide an average of 1535 tonnes of organic food waste for each day of production, and Table 1 presents BSF plants located globally that are successfully using food waste to produce BSFL for the poultry, fish, and pig production industries. AgriProtein, based in South Africa, is one of the largest commercial BSF plants, and processes 250 tonnes of food industry organic waste per day and generates seven tonnes of BSFL meal, three tonnes of bio-oil, and 20 tonnes of bio-fertiliser per day. 

**Figure 4:**
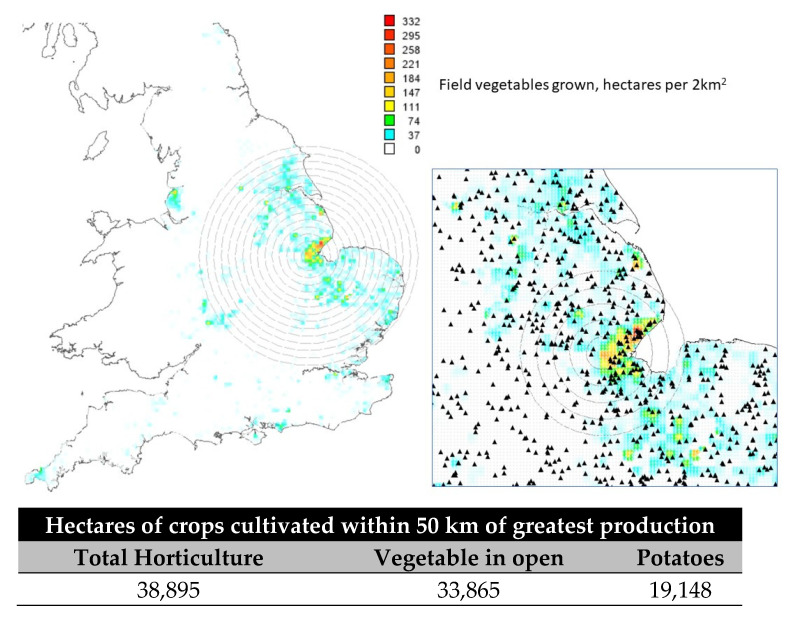
The density of field vegetables grown in England with specific focus on the regional distribution in Lincolnshire. The area of field vegetables (carrots, onions, brassicas, legumes, others) grown within 50 km of the greatest density of production is provided. The data are derived from the Defra AHS and FAME databases described in the text. The inset graphic shows the distribution of vegetable processing and trading companies within this region.

Considering this availability of food waste, the research presented has identified the biomass available from vegetable production in a regional area of the UK where vegetable production is of primary importance in supplying fresh produce to regional processors and manufacturers. The 38 food companies assessed in this study need to provide feedstock for BSFL production and obtain fresh produce for processing and manufacture. The benchmark BSFL plant data used in this study are the AgriProtein example shown in Table 1, which processes 250 tonnes of organic waste per day, and would supply six BSFL plants that would need to be established within a region where there was an optimal production, processing, and manufacturing of fresh produce. The location of food production is of importance to the development of feedstock supply chains from the food and beverage manufacturing industry for BSFL production. This is because connectivity between production, manufacturing, and feedstock supply needs to be localised to achieve the most favourable system outcome [49]. The transport of bulk bio-materials used as feedstocks is favourable for high-value materials with greater nutrient density in bulk transports [50]. Transport and distribution modelling has been used to optimise manure transportation, and strategy is critical in delivering the favoured economic outcomes for anaerobic digestion because transport costs will limit capability [51]. Prior research demonstrates that the implementation of transport strategy can reduce the economic barrier to utilising feedstocks for bioconversion to biogas, soil conditioners, and feeds, but it is the use of geographical information systems (GISs) that has been shown to incisively demonstrate the value allocation of resource flows in systems and supply chain networks [52]. The resource flow modelling that geographic information provides defines the boundaries to economic development for transporting foods, and these types of trade-off models of connectivity have been applied to anaerobic digestion capacities [53]. Figure 4 shows a GIS demonstrator of field-grown vegetable production in England, and it is clear that high-value field-grown vegetables are produced in a defined geography where soil quality is good enough to support the efficient production of high-value crops such as brassicas, salad, and root crops. The defined geography for vegetable cropping is important for this research because the Lincolnshire Region has localised production of outdoor-grown field vegetables.

Figure 4 demonstrates the localised production of field vegetables using the Defra Agriculture and Horticultural Survey (AHS) datasets that provide a resolution of the land area in a hectare production for each 2 km^2^ area. The research has been developed using data derived from open-access datasets described here that have been geocoded, so that location modelling is made possible for resource planning and respective sustainability and security assessments. MapInfo 17.02 software was used to plot geographic data using Edina Agcensus services for the Agricultural and Horticultural Survey (AHS) [45] and the fame business databases [46]. The datasets are constrained by the AHS time periods, and the data shown in Figure 4 are for 2014, the latest data available at 2 km^2^ resolution, but the land use is likely to be indicative of what is currently the case. Figure 4 enables resource mapping production with respect to potential production and location of processing and manufacturing businesses associated with agricultural production. The resource mapping uses the AHS and the Companies House FAME database to do this in a GIS (Map Info) to provide the platform resource model (Figure 4). The data for food waste from supply chains can be integrated with this resource map to indicate the amount of biomass available for bioconversion in other processes such as anaerobic digestion and BSFL production. Figure 4 shows that 33,865 ha of field-grown vegetables are within 50 km of the greatest density of field vegetable production in England. This is 27% of the total UK area cultivated for field vegetables and an estimated 0.621 × 10^6^ tonnes of vegetables using an extrapolation where the UK total field vegetable production is reported 2.333 × 10^6^ tonnes. This does not include potatoes where 19,148 hectares are grown within 50 km of the greatest density of field vegetable production, which is 18% of the total UK area cultivated for potato rotation. Using the same relationship to the total UK production of 106,000, it is 18% of the cultivated area and by the same extrapolation, 0.914 × 10^6^ tonnes of the total 5.060 × 10^6^ tonnes of UK potato production. This region’s importance is further emphasised if we consider some 5000 hectares is for protected production of horticulture and ornamental production, which is 43% of the UK total area of protected product within this 50 km radius.

When the GIS-defined data obtained in Figure 4 assumes a 10% waste in field vegetable and potato supply chains, this will provide 62 × 10^3^ tonnes of field vegetable biomass and 91 × 10^3^ tonnes of potato biomass for high-quality feedstocks in BSFL production. This does not include materials from protected production horticulture, which are highly localised with the 50 km radius tested in this research. Figure 4 demonstrates that there are 100 × 10^3^ tonnes of biomass capacity each year (with 30% contingency) to operate six industrial BSFL production sites requiring 250 tonnes of feedstock each day within the 50 km radius of the greatest field vegetable production density. Figure 4 also shows the location of vegetable processing and trading companies by their head office locators from Companies House using the FAME database. There are 289 companies within 50 km of the greatest field vegetable production density that grow or process vegetables and fresh produce, which is 9% of the UK’s total companies within these activities. Within 100 km, there are 25% of UK companies within this activity, further emphasising the case for locating BSFL production within this 50 km radius to support a connect circular economy.

**Figure 5:**
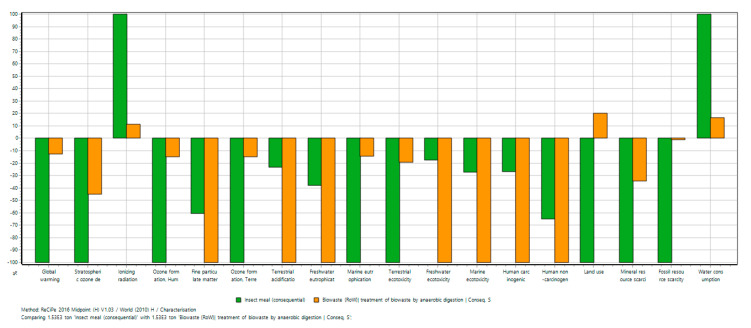
Comparison of the environmental impact of both scenarios.

We compared the environmental impact of the baseline scenario (sending food waste to anaerobic digestion) with an alternative scenario (producing insect meal with food waste and BSF and compost to be used as fertiliser). The characterised results are shown in Figure 5. For both scenarios, most of the environmental impact categories showed negative results as they both provide products with a reduced environmental impact compared to an alternative standard scenario (biogas and digestate in the first scenario; avian feed, fishmeal, and compost in the second scenario). Results showed that the BSF scenario performed better for the following environmental impact categories: global warming, stratospheric ozone depletion, ozone formation (human health), ozone formation (terrestrial ecosystems), marine eutrophication, terrestrial ecotoxicity, land use, mineral resource scarcity, and fossil resource scarcity. The current anaerobic digestion scenario performed better in the other environmental impact categories. Normalised results showed that the main environmental impact reductions were for freshwater and marine ecotoxicity and human toxicity (both carcinogenic and non-carcinogenic), mainly for the anaerobic digestion scenario.

The BSF scenario performed slightly better overall due to a reduction in environmental impacts for human health, ecosystems, and resources categories. This environmental performance could be further improved by reducing the large electricity consumption and emissions considered in the BSF scenario (12.9 kWh, 16 kg CO_2_, and 51.2 g CH_4_ per tonne of food waste treated) [48]. It must be taken into account that this LCA is just exploratory, and the scope of the analysis will be expanded in future work to account for additional processes. Differences in the products’ quality and performance were not considered between the insect meal and conventional animal feed and between the compost obtained from the BSF process and conventional compost. In conclusion, the environmental performance of using BSF to produce an insect meal and fertiliser provides a necessary circular economy solution for feeds that meets the localised procurement requirements. 

## 4. Conclusions

The localisation of BSFL production will be disruptive in current animal feed markets. The potential to produce feeds within local supply chains provides several solutions to current pressures and pinch-points in the supply of livestock feeds. The use of BSFL as a bioconversion control point provides a method of converting food wastes into high-value protein feeds. The use of BSFL as a feed is well established, but these processes of localisation provide specific benefits associated with sustainability and assurance [54]. The use of feedstocks for BSF pupae production has also identified important value chain potential such as that with the conversion of distiller grains to high protein feed for aquaculture [55]. The value of a geographic model that identifies where feedstocks are produced has been identified in the development of the BCU. The approach of using geographic information provides a food waste map that can identify where bioconversion processing can be strategically developed. The mapping approach also provides an assessment for the sustainability impact of using BSFL compared to existing feed supply chains. These maps and data are of considerable value in changing the existing feed supply chains that are limited by the global and local issues highlighted in this report.

## Figures and Tables

**Table 1:** BSFL production tested for the industrial production of animal feeds. The production facilities, location, feedstock, and production output are identified [30].

Production Facility	Location	Waste Input Type	Production Output
FORWARD	Indonesia	Market waste	0.2 tonnes of larvae/day
AgriProtein	South Africa	Food industry, restaurant and municipal organic wastes	7 tonnes of insect meal, 3 tonnes of oil and 20 tonnes of biofertiliser/day
Ento-Prise	Ghana	Market waste	About 6 kg of dried larvae/day for a feedstock of 75 kg of biofertiliser/day
Enterra Feed	Canada	Pre-consumer food waste	7 tonnes/day of protein and oil feed ingredients and 8 tonnes/day of biofertiliser
EnviroFlight	USA	Dried distillers grains with solubles	2.31 tonnes of dried BSFL meal/day
Protix	Netherlands	By-products from local distilleries, food producers, and vegetable collectors.	4.54 tonnes of BSFL/day
Alapre	Colombia	Organic waste and animal by-products	5.4 tonnes of meal/day including insect meal and compos
Entobel	Vietnam	No animal-derived ingredients	0.06 tonnes/day including insect meal, insect oil
Entofood	Malaysia	100% vegetal substrate	0.05 tonnes/day including whole insect meal, defatted insect meal, insect oil

**Table 2:** Life cycle inventory and data sources.

Process/Material	Data Source	Value
Treatment of food waste by anaerobic digestion	Ecoinvent 3 (Biowaste {RoW}|treatment of biowaste by anaerobic digestion|Conseq, S)	1525 t
Avoided avian feed	Agri-footprint (Compound feed laying hens >17 weeks/NL Economic) [47]	36.5 t
Avoided fishmeal	Ecoinvent 3 (Fishmeal, 65–67% protein, from anchovy {GLO}|market for fishmeal, 65–67% protein, from anchovy|Conseq, S) [47]	36.5 t
Avoided fertiliser	Ecoinvent 3 (Compost {GLO}|market for|Conseq, S) [47]	439 t
Water use	Water, unspecified natural origin, GB [48]	94 m^3^
Electricity use	Ecoinvent 3 (Electricity, medium voltage {GB}|market for|Conseq, S) [48]	19,798 kWh
Air emission	Water [47]	1091 t
Air emission	Carbon dioxide [48]	25 t
Air emission	Methane [48]	79 kg

## Data Availability

The datasets used in this study are available at Supplementary Materials.

## Supplementary Materials

The following are available online at https://www.mdpi.com/article/10.3390/foods10081701/s1, JAGTAP ET AL FOOD WASTE DATA.

## References

[1] FAO Global Agriculture towards 2050. 12 October 2009. http://www.fao.org/fileadmin/templates/wsfs/docs/Issues_papers/HLEF2050_Global_Agriculture.pdf.

[2] OECD Climate change and Food Systems—Climate Change and the Policy Implications for Agriculture and Fisheries. https://www.oecd.org/agriculture/topics/climate-change-and-food-systems/.

[3] FAO (2017). The Future of Food and Agriculture—Trends and Challenges. http://www.fao.org/3/i6583e/i6583e.pdf.

[4] Hall D. Brexit and Coronavirus a ‘Perfect Storm’ for Stressed East Yorkshire Pig Farmers. Hull Daily Mail, 16 February 2021. https://www.hulldailymail.co.uk/news/hull-east-yorkshire-news/brexit-coronavirus-perfect-storm-stressed-5009591..

[5] Galanakis C.M. (2020). The Food Systems in the Era of the Coronavirus (COVID-19) Pandemic Crisis. Foods.

[6] Rizou M., Galanakis I.M., Aldawoud T.M., Galanakis C.M. (2020). Safety of foods, food supply chain and environment within the COVID-19 pandemic. Trends Food Sci. Technol..

[7] FAO (2013). Faostat. FAO. http://www.fao.org/home/en/.

[8] Galanakis C.M., Aldawoud T., Rizou M., Rowan N.J., Ibrahim S.A. (2020). Food ingredients and active compounds against the coronavirus disease (COVID-19) pandemic: A comprehensive review. Foods.

[9] Galanakis C.M. (2015). Separation of functional macromolecules and micromolecules: From ultrafiltration to the border of nanofiltration. Trends Food Sci.Technol..

[10] Krzywinski M., Schein J., Birol I., Connors J., Gascoyne R., Horsman D., Jones S.J., Marra M.A. (2009). An information aesthetic for comparative genomics. Genome Res..

[11] Kloek W., Blumenthal K. (2009). Eurostat—Statistics in focus—Environment and energy. http://edz.bib.uni-mannheim.de/www-edz/pdf/statinf/09/KS-SF-09-030-EN.pdf.

[12] Jagtap S., Rahimifard S. (2017). Unlocking the potential of the Internet of Things to improve resource efficiency in food supply chains. International Conference on Information and Communication Technologies in Agriculture, Food & Environment.

[13] Capson-Tojo G., Rouez M., Crest M., Steyer J.P., Delgenès J.P., Escudié R. (2016). Food waste valorization via anaerobic processes: A review. Rev. Environ. Sci. Bio. Technol..

[14] Vandermeersch T., Alvarenga R.A.F., Ragaert P., Dewulf J. (2014). Environmental sustainability assessment of food waste valorization options. Resour. Conserv. Recycl..

[15] Arancon R.A.D., Lin C.S.K., Chan K.M., Kwan T.H., Luque R. (2013). Advances on waste valorization: New horizons for a more sustainable society. Energy Sci. Eng..

[16] Gold M., Tomberlin J.K., Diener S., Zurbrügg C., Mathys A. (2018). Decomposition of biowaste macronutrients, microbes, and chemicals in black soldier fly larval treatment: A review. Waste Manag..

[17] Galanakis C.M. (2021). Functionality of food components and emerging technologies. Foods.

[18] Gold M., Cassar C.M., Zurbrügg C., Kreuzer M., Boulos S., Diener S., Mathys A. (2020). Biowaste treatment with black soldier fly larvae: Increasing performance through the formulation of biowastes based on protein and carbohydrates. Waste Manag..

[19] Diener S., Zurbrügg C., Tockner K. (2009). Conversion of organic material by black soldier fly larvae: Establishing optimal feeding rates. Waste Manag. Res..

[20] WRAP Food Surplus and Waste in the UK—Key Facts. WRAP, 2020. https://wrap.org.uk/sites/files/wrap/Food_.

[21] Casini M., Bastianoni S., Gagliardi F., Gigliotti M., Riccaboni A., Betti G. (2019). Sustainable Development Goals indicators: A methodological proposal for a Multidimensional Fuzzy Index in the Mediterranean area. Sustainability.

[22] Market Research Future Global Animal Feed Market Research Report, By Form (Pellets, Crumbles, Mash, Others), By Species (Poultry, Ruminants, Swine, Aqua, Others)—Forecast to 2027. February 2021. https://www.marketresearchfuture.com/reports/animal-feed-market-1611.

[23] Martindale W., Swainson M., Choudhary S. (2020). The impact of resource and nutritional resilience on the global food supply system. Sustainability.

[24] Cole J.R., McCoskey S. (2013). Does global meat consumption follow an environmental Kuznets curve?. Sustain. Sci. Pract. Policy..

[25] Heuel M., Sandrock C., Mathys A., Gold M., Zurbrügg C., Kreuzer M., Terranova M. (2020). Black soldier fly larvae as a substitute for soybean in the diets of laying hens. J. Insects Food Feed..

[26] FAO How to Feed the World in 2050. 12–13 October 2009. http://www.fao.org/fileadmin/templates/wsfs/docs/expert_paper/How_to_Feed_the_World_in_2050.pdf.

[27] Smetana S., Schmitt E., Mathys A. (2019). Sustainable use of Hermetia illucens insect biomass for feed and food: Attributional and consequential life cycle assessment. Resour. Conserv. Recycl..

[28] Poore J., Nemecek T. (2018). Reducing food’s environmental impacts through producers and consumers. Science.

[29] Jagtap S., Bader F., Garcia-Garcia G., Trollman H., Fadiji T., Salonitis K. (2021). Food Logistics 4.0: Opportunities and Challenges. Logistics.

[30] Joly G., Nikiema J. (2019). Global Experiences on Waste Processing with Black Soldier Fly (Hermetia Illucens): From Technology to Business.

[31] All About Feed Update: A-Z of Insect Protein/Oil Companies. All About Feed. 6 December 2017. https://www.allaboutfeed.net/all-about/new-proteins/update-a-z-of-insect-protein-oil-companies/.

[32] Jagtap S., Garcia-Garcia G., Rahimifard S. (2021). Optimisation of the resource efficiency of food manufacturing via the Internet of Things. Comput. Ind..

[33] Jagtap S., Skouteris G., Choudhari V., Rahimifard S., Duong L.N.K. (2021). An Internet of Things Approach for Water Efficiency: A Case Study of the Beverage Factory. Sustainability.

[34] Duong L.N.K., Al-Fadhli M., Jagtap S., Bader F., Martindale W., Swainson M., Paoli A. (2020). A review of robotics and autonomous systems in the food industry: From the supply chains perspective. Trends Food Sci. Technol..

[35] Smetana S., Palanisamy M., Mathys A., Heinz V. (2016). Sustainability of insect use for feed and food: Life Cycle Assessment perspective. J. Clean Prod..

[36] Garcia-Garcia G., Woolley E., Rahimifard S., Colwill J., White R., Needham L. (2017). A methodology for sustainable management of food waste. Waste Biomass Valorization..

[37] Statista Import Value of Animal Feed in the United Kingdom (UK) from 2003 to 2018. Statista, 2021. https://www.statista.com/statistics/316185/animal-feed-import-value-in-the-united-kingdom-uk/..

[38] DEFRA Animal Feed Statistics for Great Britain—December 2017. 8 February 2018. https://assets.publishing.service.gov.uk/government/uploads/system/uploads/attachment_data/file/679900/animalfeed-statsnotice-8feb18.pdf#:~:text=Animal%20feed%20production%20was%20up%2010%25%20for%20sheep%2C,2017%20than%20December%202016%20and%2017%25%20m.

[39] Belghit I., Liland N.S., Gjesdal P., Biancarosa I., Menchetti E., Li Y., Waagbø R., Krogdahl A., Lock E.J. (2019). Black soldier fly larvae meal can replace fish meal in diets of sea-water phase Atlantic salmon (*Salmo salar*). Aquaculture.

[40] Xiao X., Jin P., Zheng L., Cai M., Yu Z., Yu J., Zhang J. (2018). Effects of black soldier fly (*Hermetia illucens*) larvae meal protein as a fishmeal replacement on the growth and immune index of yellow catfish (*Pelteobagrus fulvidraco*). Aquac. Res..

[41] Widjastuti T., Wiradimadja R., Rusmana D. (2014). The effect of substitution of fish meal by Black Soldier Fly (*Hermetia illucens*) maggot meal in the diet on production performance of quail (*Coturnix coturnix japonica*). Anim. Sci..

[42] Pietras M., Orczewska-Dudek S., Szczurek W., Pieszka M. (2021). Effect of dietary lupine seeds (*Lupinus luteus* L.) and different insect larvae meals as protein sources in broiler chicken diet on growth performance, carcass, and meat quality. Livest. Sci..

[43] Walter A., Klammsteiner T., Gassner M., Heussler C.D., Kapelari S., Schermer M., Insam H. (2020). Black Soldier Fly School Workshops as Means to Promote Circular Economy and Environmental Awareness. Sustainability.

[44] Anaerobic Digestion. Biogas Map. Anaerobic Digestion, 2020. https://www.biogas-info.co.uk/resources/biogas-map/.

[45] Edina Agcensus Maps and Data Activities. Edina Agcensus, 2019. http://agcensus.edina.ac.uk/.

[46] Bureau Van Dijk Fame Business Databases. Bureau Van Dijk, 20 December 2020. https://www.bvdinfo.com/en-gb/.

[47] Green T. Black Soldier Fly Metrics & Yields| Scale Up Production of BSF. DipTerra LLC, 31 January 2014. https://www.dipterra.com/blog/black-soldier-fly-metrics-yields-scale-up-production-of-bsf.

[48] Salomone R., Saija G., Mondello G., Giannetto A., Fasulo S., Savastano D. (2017). Environmental impact of food waste bioconversion by insects: Application of life cycle assessment to process using Hermetia illucens. J. Clean. Prod..

[49] Martindale W., Duong L., Swainson M. (2020). Testing the data platforms required for the 21st century food system using an industry ecosystem approach. Sci. Total. Environ..

[50] Pergola M., Persiani A., Pastore V., Palese A.M., D’Adamo C., De Falco E., Celano G. (2020). Sustainability assessment of the green compost production chain from agricultural waste: A case study in southern Italy. Agronomy.

[51] Esteves E.M.M., Herrera A.M.N., Esteves V.P.P., Morgado C.D.R.V. (2019). Life cycle assessment of manure biogas production: A review. J. Clean. Prod..

[52] Fernandez-Mena H., MacDonald G.K., Pellerin S., Nesme T. (2020). Co-benefits and Trade-Offs From Agro-Food System Redesign for Circularity: A Case Study With the FAN Agent-Based Model. Front. Sustain. Food Syst..

[53] Lin X., Ruess P.J., Marston L., Konar M. (2019). Food flows between counties in the United States. Environmental Research Letters. Environ. Res. Lett..

[54] Lalander C., Diener S., Zurbrügg C., Vinnerås B. (2019). Effects of feedstock on larval development and process efficiency in waste treatment with black soldier fly (*Hermetia illucens*). J. Clean. Prod..

[55] Webster C.D., Rawles S.D., Koch J.F., Thompson K.R., Kobayashi Y., Gannam A.L., Twibell R.G., Hyde N.M. (2016). Bio-Ag reutilization of distiller’s dried grains with solubles (DDGS) as a substrate for BSF larvae, Hermetia illucens, along with poultry by-product meal and soybean meal, as total replacement of fish meal in diets for Nile tilapia, Oreochromis niloticus. Aquac. Nutr..

